# Altitudinal Heterogeneity of UV Adaptation in *Phytophthora* *infestans* Is Associated with the Spatial Distribution of a DNA Repair Gene

**DOI:** 10.3390/jof7040245

**Published:** 2021-03-24

**Authors:** Yan-Ping Wang, Abdul Waheed, Shi-Ting Liu, Wen-Yang Li, Oswald Nkurikiyimfura, Yahuza Lurwanu, Zonghua Wang, Laura J. Grenville-Briggs, Lina Yang, Luping Zheng, Jiasui Zhan

**Affiliations:** 1 Key Lab for Bio-Pesticide and Chemical Biology, Ministry of Education, Fujian Agriculture and Forestry University, Fuzhou, Fujian 350002, China; 2180204012@fafu.edu.cn (Y.-P.W.); waheed90539@gmail.com (A.W.); shitingliu95@gmail.com (S.-T.L.); wenyangli96@gmail.com (W.-Y.L.); nk.oswaldo@gmail.com (O.N.); 2 Department of Crop Protection, Bayero University Kano, Kano 70001, Nigeria; ylurwanu.cpp@buk.edu.ng; 3 Fujian University Key Laboratory for Plant-Microbe Interaction, College of Life Science, Fujian Agriculture and Forestry University, Fuzhou, Fujian 350002, China; wangzh@fafu.edu.cn; 4 Institute of Oceanography, Minjiang University, Fuzhou, Fujian 350108, China; 5 Department of Plant Protection Biology, Swedish University of Agricultural Sciences, 23053 Alnarp, Sweden; laura.grenville.briggs@slu.se; 6 Department of Forest Mycology and Plant Pathology, Swedish University of Agricultural Sciences, 75007 Uppsala, Sweden; jiasui.zhan@slu.se

**Keywords:** population genomics, UV adaptation, purifying selection, ecological sustainability, climate change, DNA repair gene

## Abstract

Climate change is considered a major threat to society and nature. UV irradiation is the most important environmental genotoxic agent. Thus, how elevated UV irradiation may influence human health and ecosystems has generated wide concern in the scientific community, as well as with policy makers and the public in general. In this study, we investigated patterns and mechanisms of UV adaptation in natural ecosystems by studying a gene-specific variation in the potato late blight pathogen, *Phytophthora infestans.* We compared the sequence characteristics of radiation sensitive 23 (*RAD23*), a gene involved in the nucleotide excision repair (NER) pathway and UV tolerance, in *P. infestans* isolates sampled from various altitudes. We found that lower genetic variation in the *RAD23* gene was caused by natural selection. The hypothesis that UV irradiation drives this selection was supported by strong correlations between the genomic characteristics and altitudinal origin (historic UV irradiation) of the *RAD23* sequences with UV tolerance of the *P. infestans* isolates. These results indicate that the *RAD23* gene plays an important role in the adaptation of *P. infestans* to UV stress. We also found that different climatic factors could work synergistically to determine the evolutionary adaptation of species, making the influence of climate change on ecological functions and resilience more difficult to predict. Future attention should aim at understanding the collective impact generated by simultaneous change in several climate factors on species adaptation and ecological sustainability, using state of the art technologies such as experimental evolution, genome-wide scanning, and proteomics.

## 1. Introduction

Ongoing climate change is considered one of the biggest threats to society and nature in the 21st century [1]. It is a multiple component event associated with greenhouse gas and hazardous chemical emissions that leads to changes in ultraviolet (UV) irradiation, air temperature, precipitation, and the occurrence of extreme climatic phenomena, such as heat waves, floods, and droughts [2]. At the macroscale, climate change is associated with significant reductions in diversity, distribution and density of species, shifts in community composition, alteration of species interactions, degradation and fragmentation of ecological function, reductions in the sustainability of agricultural production, and increases in animal and plant diseases [3]. At the microscale, climate change can affect the genetic and biochemical activities of cells, the expression and evolution of genes [4], the development of traits such as body size [5] and thermal responses [6], and changes in the dispersal [7] and reproductive modes [8] of species.

UV irradiation is considered to be one of the most important environmental genotoxic agents affecting human health, ecological functions, and resilience [9]. As an important element of climate change, enhanced UV irradiation has drawn wide societal attention since the late 1970s [10]. It is mainly caused by ozone depletion associated with the rampant use of man-made chlorofluorocarbon [11]. Other meteorological parameters, such as aerosols, clouds, and surface reflectivity, accompanied by current climate change, may also indirectly influence the UV intensity intercepted at the earth’s surface. Sky observations have found that the amount of UV irradiation has increased markedly over the past decades, particularly in middle and high latitudes. For example, from 1979 to 2008, the average UVB irradiation at 32.5° N increased by ~6% [12]. Theoretical studies project that all-sky UV irradiation will continue to increase in northern latitudes in the 21st century, despite an expected recovery of the ozone layer [13]. Enhanced UV irradiation can impact human health and ecosystems, either alone or via interaction with other climate change phenomena. UV irradiation induces cyclobutene pyrimidine dimers and pyrimidine pyrimidone photoproducts in cells [14]. Formation of these covalent thymine dimers between consecutive bases disrupts the DNA structure of species. When damage is left unrepaired, it can lead to various genetic disorders, including xeroderma pigmentosum, trichothiodystrophy, neutrophil apoptosis, and skin cancers in humans [15], or inhibit photosynthesis in plants [16]. In pathogenic microbes, enhanced UV irradiation can affect conidial production, survival, dispersal, distribution, germination, and pathogenicity, which, in turn, influence disease initiation, development, and the occurrence of epidemics [17,18].

To successfully survive and reproduce, all species need to develop the ability to adapt to constant changes in environmental conditions, including enhanced UV irradiation. Both protective mechanisms that prevent or reduce the occurrence of damage to intracellular components, and repair functions that eliminate damage caused by irradiation, contribute to the UV adaptation of species [19,20]. UV damage can be prevented permanently by changing the genetic architecture of genomes, or temporally by regulating the expression of existing genes [21]. For example, plants can prevent and alleviate UV damage by changing their petiole angle, leaf shape (e.g., curling leaves and shiny wax coating), and leaf size [22], or by synthesizing particular compounds, such as anthocyanins, antioxidant enzymes, flavonoids and phenolics [22,23]. When exposed to high UV irradiation, fungal species can produce specific pigments, such as melanin [24], carotenoids and mycosporines [25] to shield themselves, delay germination, inhibit germ-tube extension [26], and/or change their ecological niches, such as invading hosts to avoid light [26,27,28]. Birds can alter eggshell color and size to minimize the risk of UV irradiation [29]. For example, eggs with a darker color can better protect embryos from UV irradiation. In addition, animals can gain protection against UV damage by migrating to ecological niches with reduced UV irradiation [28]. 

At the cellular level, adaptation to UV stress can occur through the operation of repair systems that replace damaged DNA. In animals, plants, and microbes, many DNA repair mechanisms are activated when they are exposed to intensive UV irradiation [30]. Nucleotide excision repair (NER), a versatile but highly conserved DNA repair system, eliminates a wide range of helix-distorting DNA lesions induced by environmental carcinogenic sources, including UV-induced cyclobutane pyrimidine dimers and pyrimidine pyrimidone photoproducts [14]. It excises damaged DNA and fills the gap by ligation, using the intact strand as a template [31]. The NER pathway involves ~30 genes, including radiation sensitive 23 (*RAD23*). The *RAD23* gene encodes a multifunctional protein which contains several domains, including one ubiquitin-like domain, two ubiquitin-associated domains, and one RAD4-binding domain [32]. The RAD23 protein mediates three distinct activities in the NER process: assembling the repair complex, recognizing the damaged position, and stabilizing the RAD4 protein [33,34]. 

UV adaptation varies extensively within and among species. A comparative population analysis of fitness and genomic distribution across altitudes is an effective way of understanding the patterns and mechanisms of UV adaptation in natural ecosystems. Under the same sky conditions, the amount of UV irradiation intercepted at the earth’s surface depends on the distance solar rays travel through the atmosphere and air density. This results in a ~10% increase in radiation with every 1000 m increase in altitude [35,36]. Given this, we hypothesize that organisms sampled from a higher altitude should show increased UV tolerance, and that this distribution of UV tolerance is paralleled by attitudinally related characteristics of the genomic structure of the species. 

To test these hypotheses, we compared and contrasted the sequence characteristics of the *RAD23* gene with UV tolerance in 140 *Phytophthora infestans* isolates collected from seven potato fields, varying in altitude, within China. *Phytophthora infestans* is a destructive pathogen causing late blight disease of potato and tomato. It has a worldwide distribution and can quickly adapt to environmental stresses, such as host resistance and climate change; this is likely attributable to large genomes and a high density of transposable elements [37,38]. For example, the pathogen increased by ~20% fitness after it was acclimated either at low (13 °C) or high temperature (25 °C) conditions for 200 days [39]. Germination and viability of *P. infestans* sporangia and other reproductive units can be significantly decreased even when exposed to only short periods of UV conditions [39,40]. Interestingly, even though the pathogen is generally very sensitive to UV irradiation, experimental data have shown that it can adapt to the environmental stress through both genetic and quasi-genetic mechanisms [41].

In this study, we sequenced the *RAD23* gene from diverse Chinese isolates of *P. infestans* and determined the population genetic structure, source of genetic variation, phylogenetic relationship, and evolutionary history of the gene. Combined with mycelial growth data retrieved from a previous study [41], we then compared the UV tolerance of RAD23 isoforms and evaluated the association between altitudinal polymorphisms of UV tolerance and genomic features of the *RAD23* gene among the *P. infestans* populations. We suggest that the experimental results will help predict the future evolution of species, including plant pathogens, and illuminate the possible impacts of climate change, particularly elevated UV irradiation, on plant disease in relation to ecological function and sustainability. This information might also help formulate prevention or mitigation strategies to manage ecological impacts associated with the UV-stress features of climate change.

## 2. Materials and Methods

### 2.1. Phytophthora infestans Collection 

**Figure 1:**
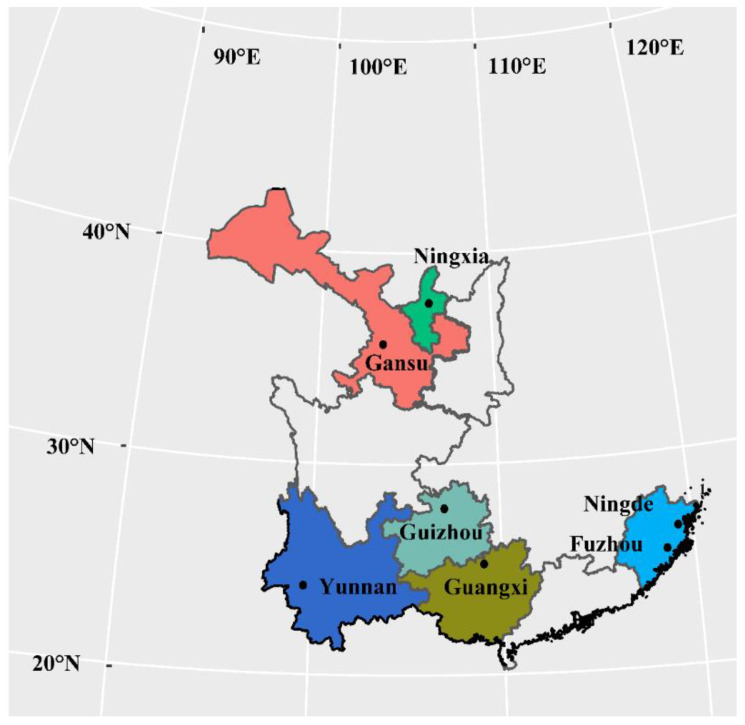
Map showing the geographical locations of the seven *Phytophthora infestans* populations sampled for this study.

*Phytophthora infestans* isolates used in the study were collected from Fuzhou, Gansu, Guangxi, Guizhou, Ningde, Ningxia, and Yunnan during 2010 and 2011. These locations vary largely in altitude (Table 1, Figure 1), climate conditions, and the frequency of potato late blight epidemics. Guizhou and Yunnan, in the southwest of China, represent the epicenters of potato late blight, where the disease occurs almost yearly, while Gansu and Ningxia, in northwest China, represent areas with climate conditions suboptimum to the development of the disease. In that part of China, potato late blight occurs only occasionally (1–2 times every 10 years). Guangxi, Fuzhou, and Ningde, in southern China, have intermediate levels of potato late blight. Isolates from the same fields were sampled from potato plants at least 100 cm apart. This was achieved by taking a piece of mycelium from a sporulating leave and inoculating it onto a rye B agar plate supplemented with ampicillin (100 μg/mL) and rifampin (10 μg/mL). The isolates were purified by sub-culturing twice and were genotyped using a combination of SSR nuclear genome assays [42,43], restriction enzyme-PCR amplification of mitochondrial genomes [44], mating type assessments [45], and a sequence analysis of functional genes (*β*-tubulin, Cox1 and Avr3a) [46], as described previously [47,48,49]. A total of 140 distinct genotypes, with 20 from each of the field populations, were selected for further study. 

### 2.2. RAD23 Sequences

*Phytophthora infestans* isolates retrieved from long-term storage were revived on rye B agar in the dark at 18 °C. The agar media was supplemented with the antibiotics ampicillin (100 μg/mL) and rifampin (10 μg/mL). After two weeks of culture, ~100 mg mycelia were harvested from each isolate, transferred into a 2 mL sterile centrifuge tube, and lyophilized overnight using a vacuum freeze dryer (Alpha1-2, Christ, Germany). The dried mycelia were ground to powder using a mixer mill (MM400, Retsch, Germany), and the DNA of the isolates were extracted using a plant genomic DNA kit, according to the manufacturer’s instructions (Promega Biotech. Co. TRANSGEN, Beijing). The extracted DNA was suspended in 50 mL ultrapure water and kept at −20 °C until use. 

A pair of specific primers (F: 5′-TCTATGATGGCTGCTAATGT-3′ and R: 5′-GTGCTTCTAGGTCCTGAC-3′) was designed from the conserved upstream and downstream of the *P. infestans RAD23* sequence (Genome number: PITG_02211; Accession number: EEY63732.1) downloaded from NCBI and used to amplify the *RAD23* sequences of the 140 *P. infestans* isolates used in this study. PCR amplifications were performed in a total reaction volume of 25 μL composed of 2.5 μL 10× HiFi Buffer II, 2.0 μL of dNTPs (10 μmol/L), 1.0 μL of forward primer (10 μmol/L), 1.0 μL of reverse primer (10 μmol/L), 16.5 μL of ddH2O, 1.0 μL HifiTaq DNA polymerase, and 1.0 μL of template DNA using a Gene Cycler^TM^ (Bio-Rad, Shanghai, China). The PCR program started with an initial denaturation step of 94 °C for 5 min; followed by 35 cycles of amplification for 30 s at 94 °C, annealing at 55 °C for 30 s, and extension at 72 °C for 60 s; and ended with a final extension step at 72 °C for 10 min. After being separated by electrophoresis on 1% agarose gels, PCR amplicons with the expected band size were purified for paired-end sequencing, according to the manufacturer’s instructions (QIAquick^®^ Gel Extraction Kit). The purified PCR amplicons were then ligated into a T5 zero vector and transformed into Trans1-T1 competent cells by heat-shock at 42 °C for 30 s (pEASY^®^-T5 Zero Cloning Kit). Colonies were randomly picked from each transformation and incubated in LB liquid media at 37 °C for 60 min under continuous shaking in 200 rpm. One colony was selected and sent to Sangon Biotech Co., Ltd. (Sangon Biotech, Shanghai, China) for sequencing using an ABI3730 automated DNA sequencer (Applied Biosystems, Foster City, CA, USA). 

### 2.3. Quantitative Real-Time PCR (qRT-PCR)

Four (A-D) of the 140 *P. infestans* isolates were randomly selected and exposed for 24 min to UV irradiation for eight days, with a 24-h interval, using an ultraviolet light C lamp (PHILIPS, wavelength = 300 nm, 30 w) placed 50 cm above the isolates. All of the UV treatments were conducted at the same time of a day. Mycelia of ~150 mg of the UV-treated isolates and their controls (without UV irradiation) were harvested and subjected to RNA extraction using a TransZol Up Plus RNA Kit, according to the manufacturer’s instructions (Promega Biotech. Co. TRANSGEN, Beijing, China). Remnant gDNA in the RNA extraction was removed by TransScript^®^ One-Step gDNA Removal, and cDNA was synthesized from the mRNA (150 ng, 50 ng/µL) by cDNA Synthesis SuperMix (Promega Biotech. Co. TRANSGEN, Beijing, China) using Anchored Oligo (dT)_18_ primers. The specific *RAD23* qRT-PCR primers (F: 5′-CTTCAGCCTCCAGTAGCACTTCTC-3′ and R: 5′-CGGACACGACATTACTGCCTTCT-3′) were designed by Primer Premier 6.0 software (Premier Biosoft International, Palo Alto, CA) from the CDS sequence of the *P. infestans RAD23* sequence (Accession: EEY63732.1) downloaded from NCBI. The qPCR was performed with a Hieff^®^ qPCR SYBR Green Master Mix (Low Rox Plus) (Yeasen Biotech Co., Ltd., Shanghai, China). Actin A gene was selected as internal control, as described previously [50], and the 2^−ΔΔCT^ method was used to compute the transcript level of the target gene relative to the Actin A gene [51].

### 2.4. Data Analyses 

Multiple sequence alignment was performed using ClustalW embedded in MEGA 7.0.21 [52]. Nucleotide composition in the *RAD23* sequences was estimated using a BioEdit Sequence Alignment Editor [53]. Nucleotide haplotypes were constructed with the PHASRE algorithm implemented in DnaSP 5.10 [54]. Isoforms were deduced by the ClustalX 2.0 program [55] and displayed using the online program ESPript (http://espript.ibcp.fr/ESPript/ESPript/ 18 August 2020). The nucleotide haplotypes were coded with letter “H”, followed by a number, and deduced isoforms were coded with “Iso”, followed by a number. Genetic variation in the *RAD23* sequences was evaluated by nucleotide diversity, haplotype diversity, and number of segregating sites using DNA Sequence Polymorphism Version 6.11.01 [56] for each of the seven sub-populations, as well as the combined total population. Homogeneity of nucleotide composition in the *RAD23* sequences, haplotype frequency, and isoform frequency among the *P. infestans* populations were evaluated by Chi-square tests [57]. Synonymous mutation (dS) and nonsynonymous mutation (dN) rates in the *RAD23* sequences were estimated using the Nei–Gojobori method [58] within MEGA 7.0.21, and the neutrality of the gene was tested accordingly by calculating dN/dS values. Recombination events within *RAD23* sequences were evaluated by a Bootscanning analysis using Simplot 3.5.1 (http://sray.med.som. jhml.edu/SCROftware/simplot, accessed on 23 August 2020).

TCS, an algorithm embedded in PopART v.1.7 (http://popart.otago.ac.nz, accessed on 26 August 2020) and used extensively to estimate gene divergence [59], was used to infer sequence genealogies through the construction of a haplotype network. Each haplotype was represented by a circle and the proportion of sequences with a particular haplotype was indicated by circle size. Steps of nucleotide substitution among the haplotypes were indicated by number of black ticks. The phylogenetic tree was reconstructed for the 140 *RAD23* sequences using the Neighbor–Joining (NJ) method [60] embedded in MEGA 7.0.21, and displayed by using Interactive Tree of Life (https://itol.embl.de/, accessed on 30 August 2020). The robustness of the phylogenetic tree was evaluated by a bootstrap test with 1000 replicates. The evolutionary distance among phylogenetic branches was computed using a Maximum Composite Likelihood method. 

UV tolerance was quantified by the growth rate of the 140 isolates treated with UVC irradiation relative to growth rate without UVC treatment. The data were reanalyzed from our previous publication [41]. Analysis of variance for the UV tolerance of the isolates and the transcript abundance data obtained from qRT-PCR was performed in SPSS [61]. Fisher’s least significant difference (LSD) test was applied to compare the UV tolerance among isolates with a particular isoform, *RAD23* expression among isolates, and *RAD23* expression between UV-treated and control isolates. Geographic parameters of pathogen collection sites, including altitude, latitude, and longitude, were measured using a mobile compass. Associations of *RAD23* sequences with the UV tolerance of their isolates, and the geographic origin of their collection sites, were evaluated by linear regression analysis [62]. 

## 3. Results

### 3.1. Sequence Variation in the RAD23 Gene of Phytophthora Infestans

The *RAD23* gene is an important component of the UV-induced DNA damage repair pathway. In this study, we analyzed the sequence variation and the spatial distribution of the gene in the *P. infestans* population to understand its role in species adaptation to UV stress. Full *RAD23* sequences were generated from all 140 *P. infestans* isolates. No introns exist in the *RAD23* gene and the gene is 1350 bp (including start and stop codon) in length, translating to a protein isoform with 449 amino acids. The mean A, C, G, and T content in the 140 sequences was 20.76%, 29.95%, 31.32% and 17.98%, respectively. Further analyses showed that base composition in the *RAD23* sequences significantly deviated from the theoretical expectation of equal proportion (*p* < 0.0001). GC content in the gene was significantly higher than its AT content and the genome average of *P. infestans* (*p* < 0.0001). 

No intragenic recombination events were detected in the *RAD23* sequences (data not shown). Point mutations (Table 2) were the only mechanism generating sequence variations. A total of nine mutation sites were detected in the 140 sequences (Table 2). Among them, three were transversions and six were transitions. The point mutations occurred exclusively in the coding regions, leaving the start and stop codon of the gene intact. As no insertions or deletions were detected in the gene, all sequences were identical in size, with the average nucleotide identity among the sequences being 99.95%. The nonsynonymous mutation rate (dN) and synonymous mutation rate (dS) were 0.1765 and 0.8063, respectively. The dN/dS value (0.219) was significantly lower than the neutral expectation evaluated by Fu’s test and the Fu and Li’s test (−2.649, *p* < 0.05), suggesting that the *RAD23* gene was subjected to purifying selection. 

**Figure 2:**
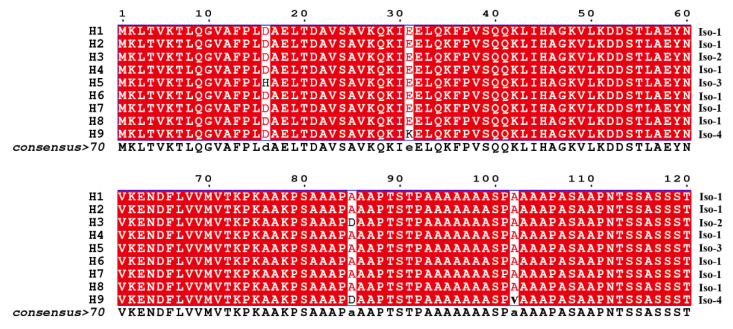
Sequence alignments of partial RAD23 isoforms deduced from nucleotide haplotypes. Different amino acids in the sequences are shown in black and shared amino acids are shown in white. The identical sequence of the isoforms in the C-terminal after the 121th amino acid is not shown. The consensus sequence (Global score > 0.70) is displayed in the last line, and the sequence alignment was performed by a ClustalW multiple approach and displayed by the online program, ESPript.

Nine nucleotide haplotypes generated by nine segregating sites were recovered from the 140 RAD3 sequences (Figure 2, Table 2 and Table 3). H2 was the most common haplotype, accounting for 57.14% of the total population. It was observed in all but the Ningde sub-populations and dominated in all but the Guangxi and Ningde sub-populations (Table 3). H4 and H8 were the second most common haplotypes, dominating in the Guangxi and Ningde sub-populations, respectively. Four isoforms were deduced from the nine nucleotide haplotypes, and the three dominant haplotypes (H2, H4 and H8), together with H1, H6, and H7, deduced to the same isoform, Iso-1 (Table 4). Iso-1 was found in all *P. infestans* sub-populations, and was the only isoform detected in Guangxi and Ningde (100%, Table 4). H3 and H9 had a point mutation from C in the consensus sequence to A at the 254th nucleotide, resulting in a change from Alanine to Aspartic acid on the 85th amino acid. H9 had an addition point mutation from C in the consensus sequence to T at the 305th nucleotide, inducing a change from Alanine to Valine in the 102nd amino acid residue. These mutations in the consensus sequence generated two rare isoforms—Iso-2 and Iso-4 (Figure 2, Table 2). The change from Aspartic acid to Histidine at the 16^th^ amino acid residue, from a base substitution of G by C at the 46th nucleotide of the consensus sequence, created another rare isoform—Iso-3, which was observed only in the sub-population sampled from Fuzhou (Table 4). Further analyses showed significant differences in haplotype (λ^2^ = 156.05, DF = 18, *p* < 0.0001) and isoform (λ^2^ = 13.62, DF = 6, *p* = 0.0342) frequencies among *P. infestans* sub-populations and UV tolerance among isoforms (*p* < 0.05) (Table 4).

Nucleotide diversity in the seven sub-populations ranged from 0.00007 to 0.00073, with a total diversity of 0.00073 in the pooled population (Table 1). Haplotype diversity in the seven field sub-populations ranged from 0.100 to 0.600, with a total diversity of 0.619 in the total population (Table 1). The highest haplotype diversity, the highest nucleotide diversity, and the richest segregating sites were found in the *P. infestans* sub-populations sampled from Guangxi, Gansu, and Ningxia, respectively (Table 1). However, the pathogen sub-population sampled from Yunnan, the site at the highest altitude, possessed the lowest number of segregating sites, the least haplotype diversity, and the least nucleotide diversity. 

### 3.2. Haplotype Network and Phylogenetic Tree of RAD23 Gene

**Figure 3:**
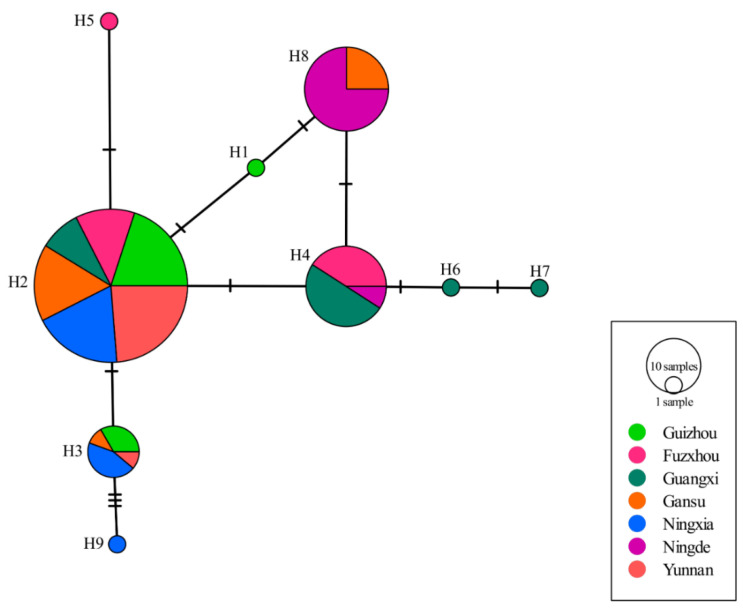
Haplotype network of *Phytophthora infestans RAD23* gene constructed by an agglomerative approach. Nucleotide haplotypes are named by the letter H, followed by a number. Each circle represents a unique haplotype, and the size of circles indicates the frequency of isolates with that particular haplotype. Each tick mark represents a step of nucleotide substitution.

The *RAD23* haplotypes diverged by a maximum of seven mutation steps, i.e., H7 versus H9 (Figure 3). Four haplotypes, including the three dominant haplotypes (H2, H4 and H8) and a rare haplotype (H1) that translated into Iso-1 (Figure 2, Table 4), formed a reticulate structure. All except H7 and H9 were two or fewer mutation steps away from the most dominant H2, and all except H3 and H9 were only one mutation step away from their adjacent haplotypes (Figure 3). Like network analysis, phylogenetic clustering using a Neighbor–Joining (NJ) approach separated the 140 *RAD23* sequences into four major branches (Figure S1). The rare H5 haplotype from a sequence in Fuzhou (F059) stood alone as a single branch, and eighty sequences from the haplotype H2 formed another independent branch with the shortest length (0.00). Nine sequences in the haplotype H3 and a sequence (pd213169) in the haplotype H9 formed the third branch with a longest length (0.0022). The fourth branch was the most diverse. It was formed by two sub-branches from 49 sequences of five haplotypes (H1, H4, H6, H7, and H8). A total 24 sequences of the haplotype H8 joined together with the sequence of haplotype H1 to form a sub-branch. The 22 sequences of the haplotype H4 clustered together with the sequences of rare haplotypes H6 and H7 to form a second sub-branch. 

### 3.3. Associations of RAD23 Sequence Characteristics in P. infestans Populations with UV Tolerance and Altitude of Collection Site of Isolates 

**Figure 4:**
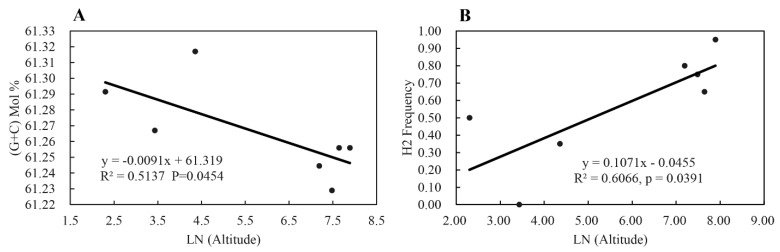
Correlation between natural logarithm transformation of altitude (m) in the collection sites and sequence characteristics (i.e., (**A**) GC content and (**B**) frequency of the dominant haplotype) of the *RAD23* gene in the seven *Phytophthora infestans* populations sampled from China.

**Figure 5:**
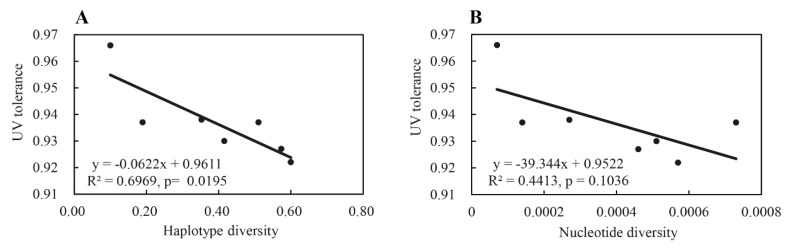
Correlation between UV tolerance and genetic variation (i.e., haplotype and nucleotide diversity) of the *RAD23* gene in the seven *Phytophthora infestans* populations sampled from China: (**A**) haplotype diversity; (**B**) nucleotide diversity.

The frequency of H2, the most dominant haplotype in the seven *P. infestans* sub-populations, was positively and significantly correlated to the altitude of sites (fields) where the pathogen sub-populations were collected, while the mean GC content of *RAD23* sequences in the pathogen sub-populations were negatively and significantly correlated to the altitude of collection sites (Figure 4). Mean UV tolerance of the seven *P. infestans* sub-populations was also negatively correlated with the mean population variation of *RAD23* sequences, but only f the correlation with haplotype diversity was significant, (Figure 5). 

### 3.4. Quantitative Real-Time PCR (qRT-PCR)

**Figure 6:**
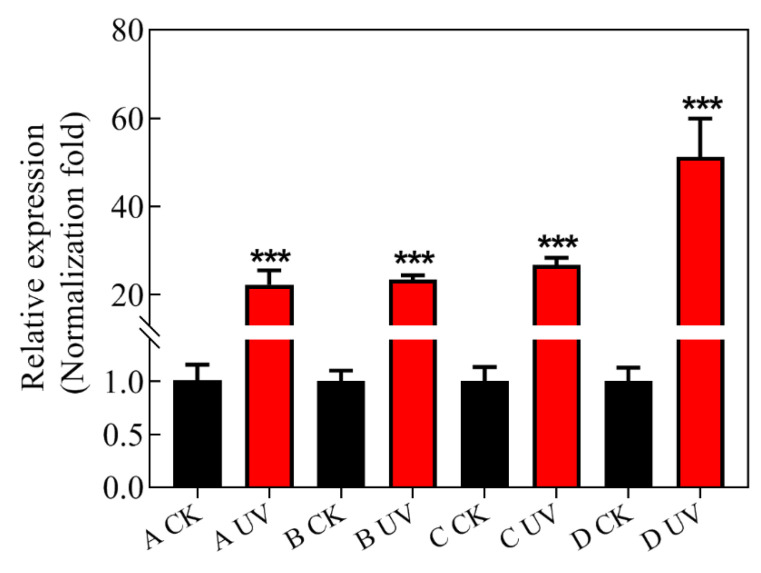
Quantitative Real-Time PCR was carried out with quantification of *RAD23* in each isolate, relative to the Actin A housekeeping gene, using the 2^−ΔΔCT^ method. The CK group consisted of control isolates without UV irradiation, and, in the UV group, isolates were exposed to UV irradiation for 20 min per day for eight days. The Actin A housekeeping gene was selected as the internal control. The experiment was repeated three times, using three independent biological replicates, and the average values are reported. The error bars represent the S.D. of the mean. *** *p* < 0.0001, compared with control groups.

*RAD23* expression was not different among the four CKs (control), i.e isolates that were not treated by UV irradiation, but *RAD23* expression was significantly different among UV-treated isolates (UVs). The abundance of *RAD23* transcripts was increased 20~50 fold in the UV treated isolates relative to CKs, indicating that the *RAD23* gene may play an important role in *P. infestans* UV adaptation (Figure 6). 

## 4. Discussion

Altitudinal heterogeneity in morphological characteristics and biochemical activities of individual organisms within the same species has been widely documented in both managed and natural ecosystems [63]. The heterogeneity has generated great impacts on species interaction, which, in turn, can alter community composition, landscape structure, and ecological function and resilience. For example, plant species living at high altitudes are generally shorter, with smaller, thicker, and more hairy leaves; they also produce less chlorophyll but more carotenoid, anthocyanin, catalase, peroxidase, superoxide dismutase, ascorbate, and peroxidase compared to those living at lower altitudes [64]. Because species encounter a gradient in UV irradiation over altitudes, with those inhabiting higher altitudes receiving stronger UV irradiation than those inhabiting lower ones [36], vertical variation in morphology and biochemistry is likely to have resulted from evolutionary adaptation of the species to UV gradients [65]. Indeed, it has been documented that plant species growing at higher altitudes with the traits described above are more tolerant to UV stresses than those occurring at lower altitudes [66]. A similar pattern of altitudinal heterogeneity in UV tolerance and species characteristics was also found in territorial animals, such as snub-nosed monkeys [67] and pathogenic microbes, such as *P. infestans* [41]. 

UV irradiation induces the formation of covalent thymine dimers that interrupt the replication and transcription of DNA [68] and may eventually lead to mutations, replication arrest, and cell death. The reduction in fitness of species associated with DNA damage can be restored when dimers are removed by nucleotide excision repair (NER). Because *RAD23* encodes a protein which is important for the assembly of the DNA repair complex [69] and recognition of UV-induced DNA damage [70] in NER, we tested the hypothesis that this gene contributes to the observed altitudinal pattern of UV adaptation in *P. infestans* and, possibly, in other species as well. Here, we provide several lines of evidence to support this hypothesis. 

Although nine haplotypes were detected in the 140 *P. infestans* isolates sampled across China, only four isoforms were identified in the populations (Table 2). Six of the nine haplotypes, including the three dominant ones (H2, H4 and H8), were deduced to the same protein isoform (Iso-1), which accounted for more than 92% of the combined population; a finding consistent with the conserved evolution of the gene [32]. The haplotype network of *RAD23* formed a reticulation structure. Due to the absence of intragenic recombination, the reticulation structure was likely generated by convergent evolution, suggesting that base substitutions occurred routinely in the *RAD23* gene. Indeed, a significantly higher GC (61.27%) than AT (38.73%) content was found in the *RAD23* sequences. The GC content is also higher than the genome average (51.0%) of *P. infestans* (Haas et al., 2009). GC is the subject target of methylation, while methylated GC (mCpG) is a hotspot of mutation [71]. Therefore, we suggest that the low genetic polymorphism in the *RAD23* sequences is likely caused by purifying selection—a hypothesis that is confirmed, since the dN/dS analysis shows a significant deviation from neutral expectations. Thus, the low genetic polymorphisms are highly unlikely to be caused only by reduced mutation rates. We further argue that the driving force of the purifying selection is UV irradiation. This argument is supported by the positive association between the frequency of the most dominant *RAD23* haplotype (H2) and altitude (i.e., UV intensity received) in *P. infestans* populations. Furthermore, the lowest number of segregating sites, haplotype diversity, and nucleotide diversity of *RAD23* sequences were found in the Yunnan sub-population, where the altitude was highest. It is also supported by the significant differences in haplotype and isoform frequency of RAD23 sequences among the *P. infestans* sub-populations sampled from areas varying in UV irradiation intensity. The positive association between H2 frequency and altitude also suggests that this haplotype can provide better protection from UV irradiation to *P. infestans*, and is, therefore, conserved due to a positive selection under enhanced UV levels at higher altitudes. 

The rich GC content in the *RAD23* sequences also points to the involvement of the gene in the UV adaptation of the pathogen. In nature, organisms display considerable variation in GC content, ranging from 25–75% of genome composition [72]. Increasingly, data have shown that this genomic feature is associated with the evolutionary adaptation of organisms to ecological or physiological conditions [73]. Because UV damage is mainly caused by mutation and cell death associated with the formation of thymine dimers, higher GC content in the genome confers a selective advantage on organisms inhabiting ecological niches subject to intense UV irradiation [74]. To ensure the retention of the RAD23 function in repairing UV damage caused to genes with important biological and biochemical functions, this UV–GC relationship predicts an enhanced GC content in the *RAD23* sequences from a higher altitude. Interestingly, we found the reverse, with a negative association between GC content in the *P. infestans* populations and the altitude of the sites at which the pathogen populations were collected. In addition to UV irradiation, thermal conditions and oxygen levels in the environment can also affect the GC content of a species genome. Thermophilic organisms tend to have a higher GC content because genes with GC triple hydrogen bonds are more stable than AT double hydrogen bonds, and many thermostable amino acids are encoded by GC-rich genes [75]. Aerobic organisms, such as aerobic prokaryotic microbes, demonstrate a tendency for higher GC content compared to anaerobic organisms [72]. Reduced air temperature and oxygen level associated with elevated altitudes are expected to select genomes with a lower GC content; the observed negative association between GC content in the *RAD23* gene and altitude in the current study may reflect the collective impact of temperature, oxygen, and UV irradiation on the genomic evolution of species. This possibility further supports the hypothesis that interactions of multicomponent changes in climate conditions would exert more complex and unpredictable influences on the functioning and resilience of ecosystems. It underlines the need for further studies that move beyond understanding individual impacts generated by changes in a single climatic factor to the synergizing impact generated by changes in multiple climactic factors.

The polymorphisms observed, namely UV tolerance among *P. infestans* isoforms, overexpression of the *RAD23* gene when the pathogen was treated by UV irradiation, and links (even though relatively weak) between sequence diversity of *RAD23* and UV tolerance in the *P. infestans* populations, provide direct and explicit evidence supporting the hypothesis that the *RAD23* gene contributes to UV adaptation of the pathogen. Among the nine haplotypes detected, H2 accounts for more than half (~60%) of the combined population, and occupies the lowest phylogenetic position. It is a maximum of two mutation steps away from the other three common haplotypes (H4, H3 and H8), and *P. infestans* isolates with this haplotype display high fitness under UV treatments. Furthermore, the haplotype increases its representation in the pathogen populations experiencing higher UV irradiation levels. Considering all of these results together suggests that the haplotype H2 is probably the wild type of the *RAD23* gene.

## 5. Conclusions

Climate change can influence every dimension of biological activity, ranging from genes to ecosystems [76]. As the most harmful and mutagenic waveband of the solar spectrum, increases in UV irradiation, together with other climate changes such as global warming, are expected to cause rapid and significant changes in the diversity and distribution of species, among-species interactions, including plant–pathogen associations, as well as in ecosystem structure [3,77,78]. Understanding the patterns and genetic and physiological mechanisms of species adaptation to UV irradiation is important for accurate predictions of the future impact of increased UV irradiation on ecosystems. In addition to physiological plasticity generated by epigenetic phenomena through the regulation of existing genes [41], the current analysis demonstrates that DNA repair genes may also contribute to the UV adaptation of *P. infestans.* The contribution of DNA repair genes in enhancing the adaptation of a species to high altitudes has also been reported in other organisms, including animals and plants [79,80,81,82]. Although these inferences indicate that many species are fully equipped to respond to UV stresses, experimental evolution combined with genomic approaches, such as passaging [39], GWAS [83]. and proteomics [84], may be required to confirm the results and to determine how quickly adaptation could occur and which other genes may be involved.

## Figures and Tables

**Table 1:** Sample size, geographic origin, and genetic diversity of *RAD23* in the seven *Phytophthora infestans* populations from China.

Pop	Sample Size	Altitude (m)	Latitude (°)	Longitude (°)	LN (Altitude)	Segregating Site Number	Haplotype Number	Haplotype Diversity	Nucleotide Diversity
Fuzhou	20	10	119.28	26.08	2.302	2	3	0.574	0.00046
Ningde	20	31	119.98	26.09	3.433	1	2	0.189	0.00014
Guangxi	20	78	108.37	22.83	4.357	3	4	0.600	0.00057
Guizhou	20	133	105.93	26.27	7.193	2	3	0.353	0.00027
Ningxia	20	1778	106.23	36.02	7.483	4	3	0.416	0.00051
Gansu	20	2088	105.72	34.58	7.644	3	3	0.511	0.00073
Yunnan	20	2676	102.72	25.05	7.892	1	2	0.100	0.00007
Total	140					9	9	0.619	0.00073

**Table 2:** Positions and types of nucleotide substitution in the nine *RAD23* haplotypes (H1-9) of *Phytophthora infestans* sampled from seven fields in China.

Positions and Types of Substitution	H1	H2	H3	H4	H5	H6	H7	H8	H9
46v *	G	G	G	G	C	G	G	G	G
75v	T	T	T	T	T	G	G	T	T
91s	G	G	G	G	G	G	G	G	A
213s	C	C	C	C	C	C	C	C	T
254v	C	C	A	C	C	C	C	C	A
305s	C	C	C	C	C	C	C	C	T
519s	A	G	G	G	G	G	G	A	G
690s	T	T	T	T	T	T	C	T	T
1137s	T	T	T	C	T	C	C	C	T

* s = transition and v = transversion.

**Table 3:** Frequency distribution of *RAD23* haplotypes in the seven *Phytophthora infestans* populations sampled from China.

Haplotypes	Guizhou	Fuzhou	Guangxi	Gansu	Ningxia	Ningde	Yunnan
H1	0.05	0.00	0.00	0.00	0.00	0.00	0.00
H2	0.80	0.50	0.35	0.65	0.75	0.00	0.95
H3	0.15	0.00	0.00	0.05	0.20	0.00	0.05
H4	0.00	0.45	0.55	0.00	0.00	0.10	0.00
H5	0.00	0.05	0.00	0.00	0.00	0.00	0.00
H6	0.00	0.00	0.05	0.00	0.00	0.00	0.00
H7	0.00	0.00	0.05	0.00	0.00	0.00	0.00
H8	0.00	0.00	0.00	0.30	0.00	0.90	0.00
H9	0.00	0.00	0.00	0.00	0.05	0.00	0.00
Total	1.00	1.00	1.00	1.00	1.00	1.00	1.00

**Table 4:** Frequency and UV tolerance of deduced isoforms in the seven *Phytophthora infestans* populations sampled from China.

Isoform	Haplotype	UV Tolerance *	Frequency						
			Guizhou	Fuzhou	Guangxi	Gansu	Ningxia	Ningde	Yunnan
Iso-1	H1-H2, H4, H6-H8	0.873a	0.85	0.95	1.00	0.95	0.75	1.00	0.95
Iso-2	H3	0.881a	0.15	0.00	0.00	0.05	0.20	0.00	0.05
Iso-3	H5	0.661b	0.00	0.05	0.00	0.00	0.00	0.00	0.00
Iso-4	H9	0.721b	0.00	0.00	0.00	0.00	0.05	0.00	0.00
Total			1.00	1.00	1.00	1.00	1.00	1.00	1.00

* UV tolerance was measured by the relative growth rate of isolates treated by UVC irradiation relative to growth rate without UV treatment. Values followed by different letters are significantly different, at *p* = 0.05.

## Data Availability

Associated *RAD23* gene sequences data generated for the 140 *Phytophthora infestans* isolates will be deposited in Genbank when the manuscript is accepted for publication.

## Supplementary Materials

The following are available online at https://www.mdpi.com/2309-608X/7/4/245/s1, Figure S1: The phylogenetic tree of Phytophthora infestans RAD23 sequences was reconstructed using a 723 Neighbor-joining (NJ) approach embedded in the MEGA 7.0.21 program and displayed and annotated using 724 the online tool Interactive Tree of Life (https://itol.embl.de/, accessed on 23 August 2020). The bootstrap values were calculated from 1000 replications and are represented by red circles at the middle of the tree graph.
