# Supplementary material for: Altitudinal Heterogeneity of UV Adaptation in Phytophthora infestans Is Associated with the Spatial Distribution of a DNA Repair Gene
Source: J Fungi (Basel). 2021 Mar 24;7(4):245. doi: 10.3390/jof7040245 (PMC8064308; doi:10.3390/jof7040245)
Supplement: Supplementary file 1 [file jof-07-00245-s001.pdf]

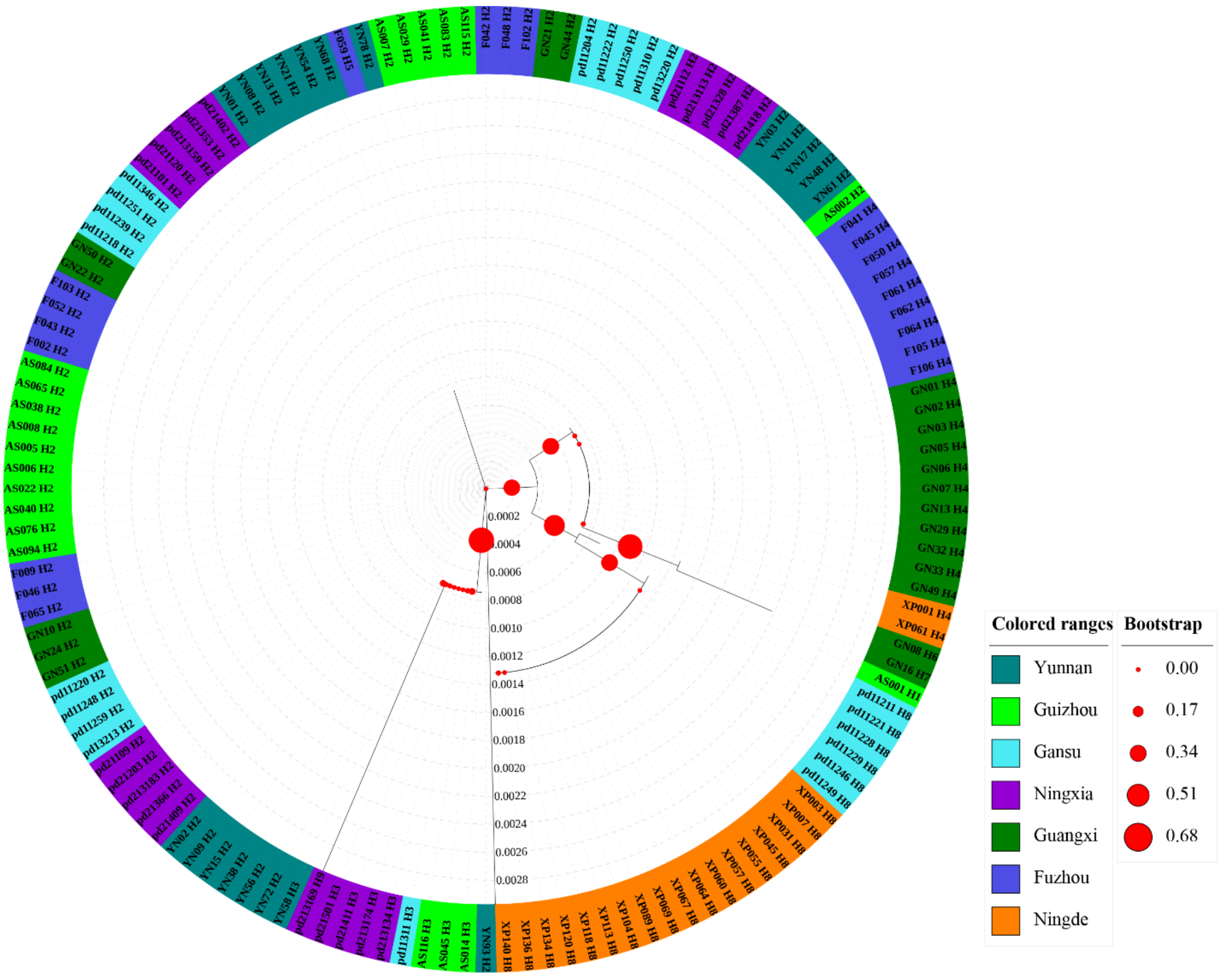

Figure S1. The phylogenetic tree of *Phytophthora infestans RAD23* sequences was reconstructed using a Neighbor-joining (NJ) approach embedded in the MEGA 7.0.21 program and displayed and annotated using the online tool Interactive Tree of Life (https://itol.embl.de/). The bootstrap values were calculated from 1,000 replications and are represented by red circles at the middle of the tree graph.
